# Glycofullerenes as non-receptor tyrosine kinase inhibitors- towards better nanotherapeutics for pancreatic cancer treatment

**DOI:** 10.1038/s41598-019-57155-7

**Published:** 2020-01-14

**Authors:** Maciej Serda, Katarzyna Malarz, Anna Mrozek-Wilczkiewicz, Marcin Wojtyniak, Robert Musioł, Steven A. Curley

**Affiliations:** 1 Institute of Chemistry, University of Silesia in Katowice, Katowice, 40-006 Poland; 2 Institute of Physics and Silesian Center for Education and Interdisciplinary Research, University of Silesia in Katowice, 75 Pułku Piechoty 1A, Chorzów, 41-500 Poland; 3 CHRISTUS Trinity Mother Frances Oncology Institute, Tyler, TX 7570 USA

**Keywords:** Cancer, Chemical biology, Drug discovery, Chemical biology, Medicinal chemistry, Nanomedicine, Nanoscale materials

## Abstract

The water-soluble glycofullerenes **GF1** and **GF2** were synthesized using two-step modified Bingel-Hirsch methodology. Interestingly, we identified buckyballs as a novel class of non-receptor Src kinases inhibitors. The evaluated compounds were found to inhibit Fyn A and BTK proteins with IC_50_ values in the low micromolar range, with the most active compound at 39 µM. Moreover, we have demonstrated that formation of protein corona on the surface of [60]fullerene derivatives is changing the landscape of their activity, tuning the selectivity of obtained carbon nanomaterials towards Fyn A and BTK kinases. The performed molecular biology studies revealed no cytotoxicity and no influence of engineered carbon nanomaterials on the cell cycle of PANC-1 and AsPC-1 cancer cell lines. Incubation with the tested compounds resulted in the cellular redox imbalance triggering the repair systems and influenced the changing of protein levels.

## Introduction

Pancreatic cancer is an extremely aggressive type of cancer with a poor prognosis and a five-year survival rate of less than 7%^1^. Moreover, only about 20% of patients are eligible for surgical resection, the only potentially curative treatment^2^. Chemotherapeutic approaches, which are often based on gemcitabine, are still present in the clinic as the adjuvant therapy of choice for resected pancreatic cancer. The novel cytotoxic combination strategies and nanoformulations which include *Folfirinox* and *nab-paclitaxel* are being developed intensively, but these treatments have their own shortcomings and their therapeutic effects are disappointing^3^. Nanomedical strategies which include metallofullerene-based (Gd@C_82_(OH)_22_) inhibitors of matrix metalloproteinases (MMPs)^4^, photothermally active nanoparticles^5,6^, and several nanodelivery systems^7,8^ are also being presented in attempts to improve survival in pancreatic cancer patients. At the same time, the pharmaceutical industry has focused significant attention around the use of selective tyrosine kinase inhibitors as anti-cancer agents^9^. The EGFR kinase inhibitor Erlotinib has been approved by FDA for use in combination with gemcitabine for locally advanced or metastatic pancreatic cancer^10^. Recently, some reports have suggested that inhibition of non-receptor intracellular Src kinases inhibits growth and metastasis of human pancreatic carcinoma in murine models, and is followed by synergetic effects in combination therapy with gemcitabine^11^. In order to overcome the barriers to standard chemotherapeutics, we have been exploring the use of [60]fullerene derivatives as novel EPR-targeted nanotherapeutics in *in vitro* and *in vivo* models^12,13^. Previously, we have reported the creation of photoactive and highly water-soluble glycofullerene **GF2** (termed ‘Sweet-C_60_’) that predominantly accumulates in the nucleus of pancreatic stellate cells (PSCs) and is inherently non-toxic even in high concentrations (above 1 mg/mL)^14^.

## Results and Discussion

**Figure 1:**
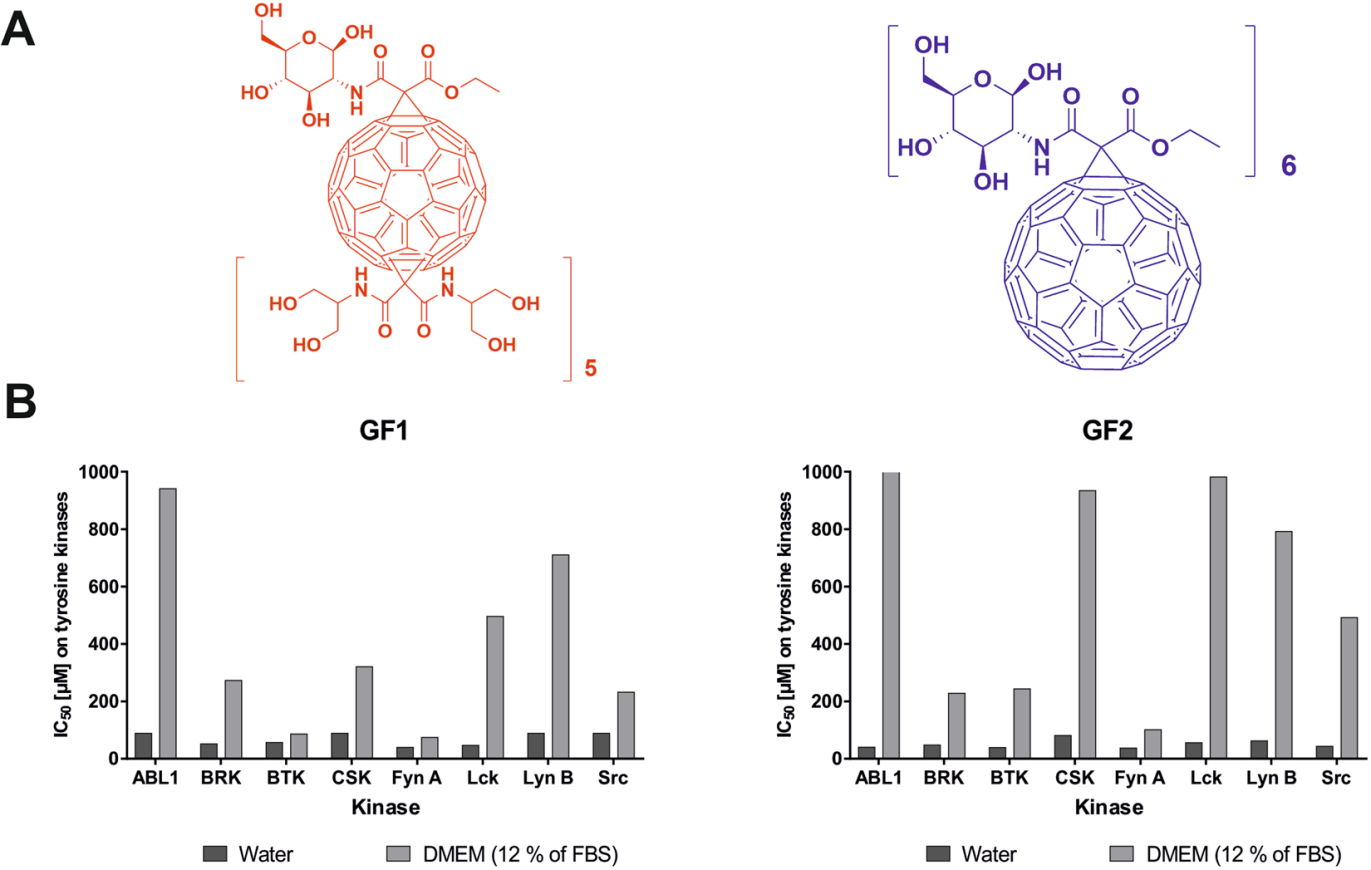
(**A**) Structures of **GF1** and **GF2** glycofullerenes; (**B**) Inhibitory activity of glycofullerenes on a panel of non-receptor tyrosine kinases.

Based on the above background, we have synthesized two glycofullerenes: **GF1** and **GF2** (see Fig. 1A) containing *D*-glucosamine fragments as glycosyl donors in the central core, including full characterization of all intermediates through MALDI-TOF mass spectroscopy, ESI-mass spectroscopy, ^1^H- and ^13^C-NMR, DLS, and zeta potential measurements (see Supporting Information, Figs. S1–S5). The Src kinase inhibitory properties for the synthesized glycofullerenes have been thoroughly evaluated, and detailed studies on protein corona formation around the glycofullerenes and its influence on selectivity and inhibitory Src kinase activity (see Fig. 1B and Table 1) have been carried out. The enzymatic studies of [60]fullerenes have been extended to time-dependent mRNA and protein expression analysis for Fyn A and Lck kinases which was performed on two human pancreatic cancer cell lines: PANC-1 and AsPC-1. The influence of glycofullerenes on cell cycle regulation as well as induction of autophagy in pancreatic cancer cell lines have also been investigated. To the best of our knowledge, this is the first work on the use of [60]fullerene nanomaterials as selective non-receptor tyrosine kinases inhibitors.

**Table 1:** Inhibitory activity of glycofullerenes **GF1** and **GF2** on non-receptor tyrosine kinases panel performed in water and in DMEM with 12% of FBS.

Compound	IC_50_ [µM] on tyrosine kinases							
	ABL1	BRK	BTK	CSK	Fyn A	Lck	Lyn B	Src
GF1 in water*	>88.850	51.533 ± 9.329	56.864 ± 12.439	>88.850	39.982 ± 2.665	46.645 ± 3.554	>88.850	>88.850
GF1 in DMEM*	940.91 ± 149.711	272.768 ± 46.646	86.184 ± 6.664	320.302 ± 53.310	74.189 ± 20.88	496.224 ± 20.880	710.351 ± 139.494	231.897 ± 46.202
GF2 in water*	40.146 ± 2.028	47.851 ± 3.244	38.929 ± 3.244	>81.103	37.307 ± 3.244	55.961 ± 11.760	62.044 ± 1.216	43.796 ± 1.216
GF2 in DMEM*	>1000	228.305 ± 60.827	243.714 ± 38.118	933.901 ± 229.16	100.973 ± 17.437	981.346 ± 221.4112	791.565 ± 238.037	491.484 ± 156.123
Dasatinib^21^**	0.0031 ± 0.0005	0.005 ± 0.0005	0.003 ± 0.0007	0.0013 ± 0.0003	0.0016 ± 0.0008	0.0023 ± 0.0007	0.0012 ± 0.0001	0.0009 ± 0.0001

^*^The final concentration of solvents (water, DMEM or DMSO) is 5%.

**Dasatinib was dissolved in DMSO.

Previously, we have demonstrated that fluorescently-labelled [60]fullerene derivatives were able to extravasate more into orthotopic murine breast tumor tissues than into the contralateral mammary fat pad^13^. In this study we propose that glycosylation of the [60]fullerene core will promote cancer targeting of engineered carbon nanomaterials due to overexpression of glucose transporter membrane proteins (GLUTs) in several cancers, including pancreatic adenocarcinoma^15^. Interestingly, we have performed qRT-PCR experiments on two human pancreatic cancer cell lines, showing that AsPC-1 cells present 10 fold higher levels of *GLUT-1* mRNAs in comparison to PANC-1 cells (see Supporting Information, Fig. S9). We decided to use diserinol malonate (possessing four hydroxyl group) as the second addend attached to our [60]fullerene scaffold. Our previously published experiments describing molecular biology impact of compound C_60_-ser have reported its highly water-soluble properties and lack of *in vitro* cellular and *in vivo* murine toxicity^16,17^. Moreover, the metal chelating abilities of hydroxyfullerenes have been recently explored, and they should be considered as an important factor modulating their interactions with cellular targets, such as enzymes^18^.

To synthesize the aforementioned structurally diverse glycofullerenes, the modified Bingel-Hirsch methodology was applied. The presented synthetic procedure enables the creation of a broad spectrum of [60]fullerenes and allows generation of structurally more complicated systems thanks to high synthetic accessibility of functionalization malonates. The [60]fullerene nanomaterials **GF1** and **GF2** were synthesized in the two-step cyclopropanation reactions, in which [60]fullerene derivative **3** was used as the substrate for the final products (see Supporting Information, Fig. S1). The [60]fullerene hexakis-adduct with *T*_*h*_-symmetry (**GF1**) bearing two or more different peripheral functional subunits was obtained in the second Bingel reaction from monoadduct **3** as a starting material. The glycofullerene **GF2** was obtained as a mixture of regioisomers without further purification, and its full chemical characterization had been published earlier^14^. The MALDI-TOF mass spectrum of peracetylated **GF1** is shown in Fig. S7 (Supporting Information), showing characteristic molecular peaks for hexakis-adduct at 3412 Da. A molecular peak ion from the deprotected sodium adduct **GF1** is presented at 2273.2 Da, showing also small signals from pentakis isomers at 2026.1 Da (Supporting Information, Fig. S8). As can be seen in Fig. S4 (Supporting Information), glycofullerene **GF1** seems to form aggregates at around 100 nm and shows a surface charge of −26.7 mV based on DLS and zeta potential measurements. Mackeyev *et al*. reported experiments conducted for the water-soluble C_60_ derivatives, especially those with diserinol units, suggested that those derivatives form highly-dynamic aggregates, sized between 100–300 nm in diameter, which exist in equilibrium with free, hydrated fullerene molecules (based on SEM studies)^19,20^. Moreover this equilibrium is connected with addition of organic solvents and ionic strength (salts), which complicates understanding of protein corona formation. In addition, to better understand formation of glycofullerene aggregates, we performed AFM studies using the Si (001) substrate. As one can notice, the substrate was extremely flat, with the roughness, calculated as root mean square (RMS), of only 72.8 pm (Fig. S6A). The topography obtained from the AFM shows two types of agglomerates for both glycofullerenes **GF1** and **GF2**. In the case of **GF1**, the first type consist of separate round-like objects, with the average diameter equal to 19.24 nm diameter on average (Fig. S6B), b) larger agglomerates with average sizes of 270–300 nm (Fig. S6C). The [60]fullerene derivative **GF2** shown in Figs. S6D-E was found to be very similar to the previous sample. We have observed two primary types of agglomerates– smaller and larger ones. The primary agglomerates were found to have 17.68 nm diameter on average and heights around 10 nm, while the larger ones were around 250–280 nm in diameter and height of 400 nm consisting of smaller objects. The smaller observed objects were often asymmetric, suggesting that they are formed of even smaller parts (separate fullerenes). Unfortunately, due to the size of the AFM tip limiting the image resolution, we were not successful in observing those fullerenes. The larger objects however, could be easily distinguished, and were formed of smaller agglomerates. Due to protein corona formation on the surface of synthesized glycofullerenes, we were also interested in the samples dissolved in the DMEM containing FBS, however we have found a huge difficulties in the measurements of those samples. The typical result for the FBS containing sample is shown in Fig. S6E. One can notice very large structures (tens of µm in diameter), with branch- or leaf-like morphology. Unfortunately, the structures were so large, that no glycofullerenes were detected. We have performed a number of experiments with diluted samples trying to limit the salt content. The crystal sizes could be diminished, but still no aggregates were visible. This could be due to the fact that those aggregates could serve as perfect base for the salt crystals to growth from, thus hiding the agglomerates inside.

With a synthetic methodology in hand, we have tested the [60]fullerene nanomaterials as potential inhibitors of non-receptor tyrosine kinases including the ABL1, BRK, BTK, and Src family kinases (CSK, Fyn A, Lck, Lyn B and Src). As depicted in Table 1, the **GF1** compound exhibits inhibitory properties in micromolar ranges, modulating the catalytic activity of Fyn A and Lck proteins (IC_50_ parameters 39.982 and 46.645 µM, respectively). Interestingly, almost no inhibitory activity was observed for the ABL1, CSK, Lyn B, and Src kinases. The **GF2** compound shows a relatively high inhibitory activity pattern for Src family kinases, with the exception of the CSK protein, and it also presents the lowest value of the IC_50_ parameter for the Fyn A enzyme (37.307 µM). Additionally, this carbon nanomaterial exhibits a high potential of inhibition against the ABL1, BRK, and BTK kinases. Due to the lack of water-soluble nanomaterials with inhibitory activity on the Src kinases, a small molecular inhibitor dasatinib has been used as a reference compound in these studies. It presents a nanomolar activity on all tested tyrosine kinases, but possesses a low selectivity profile within the tested subgroup of kinases, as well as cytotoxic effects on a normal cell line (NHDF)^21^.

It has been previously reported by experimental and computational approaches that at least 30 proteins may interact with [60]fullerenes, including human serum albumins, lysozyme, and HIV proteases^22^. Based on plethora of literature reports describing formation of protein corona on the surface of carbon nanomaterials, we have decided to carry out inhibition experiments using a cellular medium DMEM containing 12% of *fetal bovine serum* (FBS)^23^ as a solvent. The DLS measurements (see Supporting Information, Fig. S5) on the **GF1** glycofullerene confirm that it forms aggregates of around 100 nm that are being rapidly reorganized in the presence of FBS into three different subgroups: 8, 30, and 195 nm. The final results of inhibitory activity of C_60_-FBS complexes are depicted in Table 1. As expected, the protein corona formed on the surface of glycofullerenes have modified the landscape of their reactivity changing it to higher values of IC_50_ parameters (Fig. 1B). What is more appealing, the selectivity profiles of **GF1** and **GF2** have been significantly altered. For the **GF1** compound, it is inactive towards almost all the tested tyrosine kinases with the exception of the Fyn A and BTK kinases with IC_50_ parameters below 100 µM. The **GF2** glycofullerene shows the greatest inhibitory activity on Fyn A kinase (IC_50_ parameter around 100 µM) with no activity on ABL1, CSK, Lck, and Lyn B. Although molecular docking studies should also be performed in order to analyze the C_60_-kinases interactions, it can be assumed that glycofullernes do not interact with the active site of the aforementioned kinases but rather show an allosteric type of inhibition.

To better understand interactions of fullerene nanomaterials with serum proteins, as well as their inhibitory activities on non-receptor kinases, we incubated compound **GF2** at two different concentrations (1 and 2 mg per mL) with 10% and 100% of FBS. The results are depicted on the Fig. S11 in the Supporting Information. Based on the densitometric analysis, we could confirm that the intensities of protein bands around 60, 110 and 160 kDa are changed (in comparison to 100% of FBS alone), what is related to the concentration of [60]fullerene nanomaterial. Interestingly, when **GF2** is incubated with FBS at higher concentration (2 mg/mL), the most increased band is that around 65 kDa (it could be associated as *bovine serum albumin* band, molecular weight around 66 kDa). In contrast for the lower concentration studies, one could observe marked increasement of bands for the higher mass proteins (110 and 160 kDa). This phenomenon is also observed when the 10% percent FBS studies, and also confirmed by other reports in which different nanomaterial concentrations could affect formation of different protein coronas^24^. The obtained experimental results support the hypothesis of protein corona formation around synthesized glycofullerene **GF2**, but further mass spectrometry followed bioinformatic analysis should be carried out to identify the exact binding proteins.

**Figure 2:**
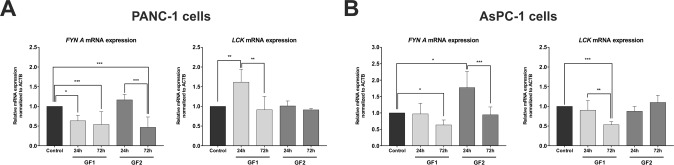
qRT-PCR analysis of expression of *FYN* and *LCK* in PANC-1 (**A**) and AsPC-1 cells (**B**) after 24-hour and 72-hour incubation with glycofullerenes (1 mg/mL). The results are shown as the mean ± SD of four independent measurements, each in triplicate. Data were analyzed using one-way ANOVA with Bonferroni’s post-hoc test: *p < 0.05, **p < 0.01, ***p < 0.001 compared to the control and respective compounds.

**Figure 3:**
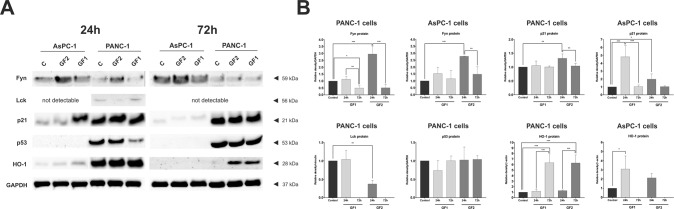
The effect of glycofullerenes (1 mg/mL) on the expression of Fyn, Lck, p21, p53, and HO-1 in PANC-1 and AsPC-1 cells (**A**). Densitometric analyses of these proteins were normalized to GAPDH, vinculin, or β-actin. The results are the mean ± SD of three independent experiments. Statistical differences were analyzed using one-way ANOVA with a Bonferroni’s post-hoc test: *p < 0.05, **p < 0.01, ***p < 0.001 compared to the control group and respective compounds (**B**).

The interesting ability of glycofullerenes to inhibit tyrosine kinases *in vitro* prompted us to evaluate their mechanism of action at the cellular level. For this purpose, we have performed an analysis of the transcript and the protein levels of Fyn and Lck in the two human pancreatic cell lines after treatment with these glycofullerenes. The results of these experiments are presented in Figs. 2 and 3. The qRT-PCR analysis revealed that the **GF2** compound caused a significant increase in the expression of *FYN* after 24-hour incubation in AsPC-1 cells (Fig. 2B). In case of PANC-1 cells, we have not observed any statistically significant changes after 24-hour of incubation with **GF2** (Fig. 2A). In turn, after the next 48 hours we have observed a significant decrease in expression of *FYN* in both tested cell lines. For the **GF1** compound, we have noticed a decrease in the level of *FYN* in PANC-1 cells after 24-hour incubation. Then, after the next 48 hours of incubation, the decreased level of *FYN* are maintained in both cell lines. We have observed similar changes in protein levels in both pancreatic cell lines (Fig. 3B). In detail, after 24-hours of treatment with **GF2** we observed an almost 3-fold increase in the expression of Fyn in PANC-1 and AsPC-1 cells. Then, after the next 48 hours, protein levels were dramatically decreased in PANC-1. For AsPC-1, we have recorded similar effects but to a lesser extent. Similarly, the decreased expression of Fyn has also been registered for the **GF1** compound in PANC-1 cells. The time-dependent changes in the expression of Fyn at the cellular level could be explained by several factors. The intensified accumulation of this protein may be the result of enhanced transcription of the *FYN* gene. In addition, this effect may be due to the interaction of glycofullerenes with a kinase, which may contribute to the inhibition of activity and blocking Fyn protein phosphorylation. In turn, the decrease in the level of protein over time may be indirectly related to its degradation. It should be emphasized that both carbon nanomaterials have shown the highest inhibitory potential against Fyn kinase in both water and DMEM medium. In the case of the *LCK* gene, we have observed the reverse behavior after treatment with carbon nanomaterials (Fig. 2). Only the **GF1** compound affects changes in the *LCK* level in pancreatic cells. After 24 hours of treatment, we observed a significant increase in the level of this gene in PANC-1, while after 72 hours we noticed the opposite situation, namely the level of *LCK* had decreased in both cell lines. It is worth mentioning that the **GF1** compound has exhibited greater potential for inhibiting Lck kinase (Table 1). However, on the protein level we have recorded a dramatic decrease after 24-hour incubation with **GF2** in PANC-1 cells (Fig. 3B). In other cases, the Lck protein level was undetectable with the use of the Western Blot method.

**Figure 4:**
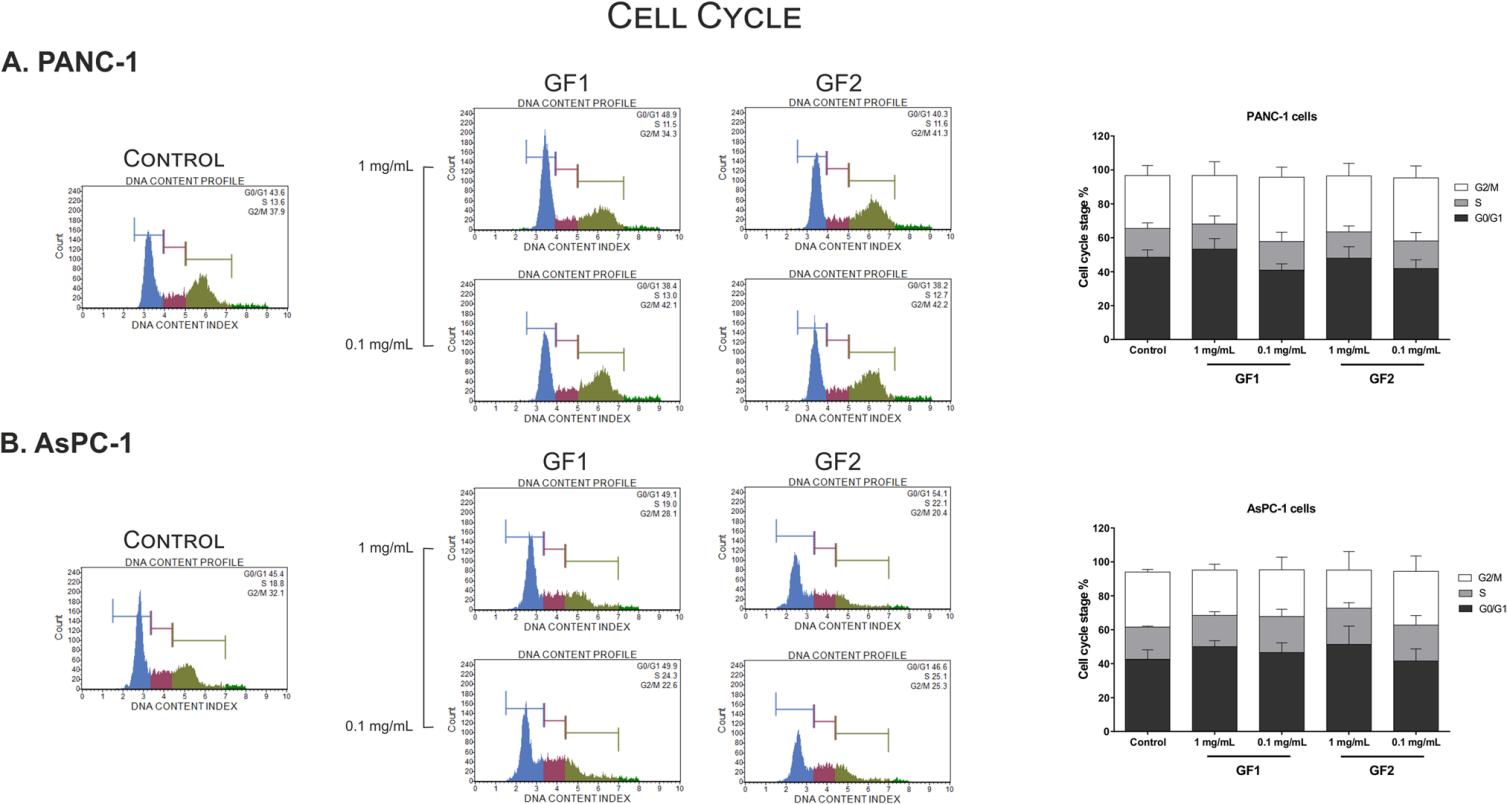
Influence of glycofullerenes on the cell cycle progression in pancreatic cell lines. The representative histograms from flow cytometry show cell cycle distribution after 24-hour incubation with glycofullerenes (0.1 and 1 mg/mL) in PANC-1 (**A**) and AsPC-1 (**B**) cells. The graph shows the mean ± SD percentage of cells in the G0/G1, S, and G2/M phases of the cell cycle from three independent experiments.

The Fyn and Lck kinases belong to the Src family that plays a crucial role in the signaling networks involved in the regulation of various cellular processes, such as proliferation, cell cycle progression, adhesion, migration, invasion, and cell survival^25–28^. With this in mind, we have determined the influence of the tested glycofullerenes on the expression of p21 and p53 proteins that are associated with regulation of the cellular proliferation and programmed cell death induction (Fig. 3). Interestingly, we have not observed any changes in p53 expression in PANC-1. It is noteworthy that AsPC-1 cells possess two mutated p53 alleles, resulting in absence of both transcript and protein^29^. On the other hand, the western blot analysis has revealed influence of glycofullerenes as kinase inhibitors on upregulation of p21 protein. In general, we have observed a significant increase of p21 levels in AsPC-1 cells after 24-hour incubation with both glycofullerene compounds. We have also recorded increased p21 expression in PANC-1 cells after a 24-hour treatment with **GF2**. These observations are consistent with the previous report on the direct relation between inhibition of Fyn and activation of p21 in pancreatic cells^26^. In addition, Jiang *et al*. demonstrated that inhibition of Fyn activity contributes to phosphorylation of hnRNP E1 through activation of p21, which affects alternative splicing of integrin β. It is noteworthy that integrins play an important role as receptors in the invasion and metastasis of pancreatic cancer cells^30^. On the other hand, the fact that synthesized glycofullerenes did not affect cell cycle arrest in pancreatic cells is surprising (Fig. 4).

**Figure 5:**
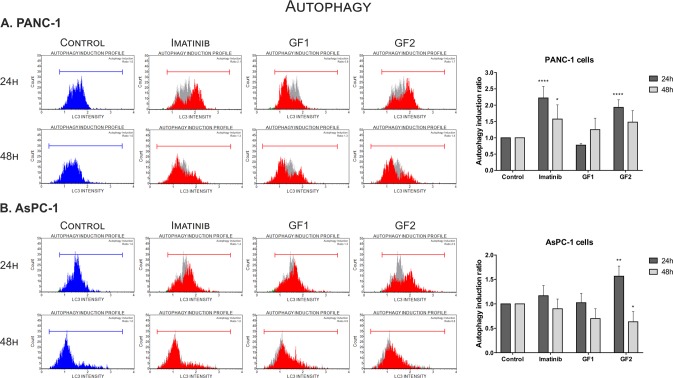
Influence of glycofullerenes (1 mg/mL) on autophagy induction in PANC-1 (**A**) and AsPC-1 (**B**) cells. The histograms show the autophagy induction ratio that was calculated by comparison of fluorescence from control cells (blue or gray peak) to the tested compounds (red peak). As a positive control, Imatinib (25 μM) was used. Data were analyzed using one-way ANOVA with Bonferroni’s post-hoc test: *p < 0.05, **p < 0.01, ***p < 0.001 compared to the control (untreated cells).

We next decided to determine the effect of these carbon nanomaterials on the induction of cell death *via* autophagy. Interestingly, it had been described earlier that NanoC_60_ nanomaterial had been chemo-sensitizing in doxorubicin-resistant HeLa cells *via* autophagy mediated pathways^31^. Here, we have performed tests based on staining with anti-LC3 Alexa Fluor555 conjugated antibody indicative of autophagy and fluorescence measurement using flow cytometry. The results are presented in Fig. 5. In general, we observed a strong effect on the formation of autophagosomes and autophagy induction after a 24-hour treatment with **GF2** in both pancreatic cancer cell lines that were tested. We have also calculated the autophagy induction ratio by comparison of **GF2** to control cells, which was 1.9 for PANC-1 and 1.5 for AsPC-1 cells.

Interestingly, these effects were abolished after 48 hours. The possible explanation of these fluctuations could be activation of the cellular repair pathways, following an earlier cellular stress response. Luo *et al*. demonstrated that autophagy may be induced by ROS formation and upregulation of p21 protein^32^. Additionally, the sustained high level of intracellular ROS may cause activation of p38 MAPK kinase and its downstream pathways that are involved in cell growth arrest and apoptosis induction specifically in response to oxidative stress^33,34^. With this in mind, we decided to determine the effect of the tested glycofullerenes on the generation of ROS. In this purpose, we conducted a series of experiments with different incubation times (9, 12, 24 h) with **GF1**, **GF2** and their combination with ROS quencher - Neocuproine (NCP) in PANC-1 and AsPC-1 cells. The concentration of ROS were measured using the CellROX fluorescence assay according to protocols established by our group for small-molecule compounds^35^. In general, we observed an increase of ROS levels after incubation with the tested glycofullerenes in both cell lines (see Supporting Information, Fig. S10**)**. The highest increase was observed after 9 h incubation in PANC-1 cells (about 130%) – Fig. S10A. Then, in subsequent incubation time points with **GF1** and **GF2**, we noticed a slight decrease to 110% in comparison to untreated control. Similarly effect, but to a lesser extent, was observed in AsPC-1 cells (Fig. S10B). Moreover, the addition of NCP, which is known ROS scavenger and glycofullerenes resulted in decrease ROS concentration below basal level in pancreatic cells. The upregulation of autophagy by carbon nanomaterials may also represent an interesting tool to induce cell death in cancer cells.

Heme oxygenase (HO-1) is another oxidative stress marker that may be activated by similar stimuli including polyhydroxylated fullerenes^36^. Under normal conditions, HO-1 expression is low in the majority of cells and tissues. Some reports have demonstrated that upregulation of HO-1 is regulated by signaling pathways, in which MAPK kinases play a key role^37,38^. With this in mind, we have evaluated HO-1 levels after 24- and 72-hour treatment with glycofullerenes (Fig. 3B). We noticed an almost 7-fold increase in the expression of HO-1 in PANC-1 cells after 72 hours in both compounds. However, a shorter incubation has no significant effect on the protein level in this cell line. Contrarily, we recorded a significant increase for **GF1** after 24-hour incubation in AsPC-1 cells. With **GF2** we observed a very slight increase in HO-1 levels. After a 72-hour incubation, the protein level was undetectable with the use of the Western Blot method, which may indicate that redox balance has been recovered.

To conclude, this novel report indicates glycofullerene-based inhibitors of non-receptor tyrosine kinases are able to selectively modulate the activity of Fyn A and BTK/Lck proteins. Positive influence of serum proteins may suggest that formation of fullerene-protein corona is an important factor in the process of inhibition. The performed SDS-PAGE electrophoresis studies confirmed formation of protein corona on glycofullerene **GF2**. Interestingly, the synthesized carbon nanomaterials have been found to be non-toxic, neutral for the pancreatic cancer cell cycle, but induces autophagy and disrupts redox balance. This work opens a new avenue in [60]fullerene-protein biomedical studies and it reflects a significant advancement in developing pancreatic cancer nanotherapeutics. Further studies explaining mechanisms of inhibition, especially for the Fyn A and BTK/Lck kinases, should be carried out with particular emphasis on determination of the K_m_ and V_max_ parameters that are crucial for understanding this type of inhibition.

## Methods

### Synthesis of glycofullerenes **GF1** and **GF2** and their malonic acid precursors

All compounds used were reagent grade or better, solvents were used as received unless otherwise specified. The following reagents were used as received: C_60_ (99.5+%, SES Research, USA), *D*-glucosamine hydrochloride (Sigma Aldrich), DBU (1,8-diaza-bicyclo[5.4.0]undec-7-ene, Sigma Aldrich), ethyl hydrogen malonate (Sigma Aldrich), CBr_4_ (Sigma Aldrich), 2-amino-1,3-propanediol (AK Scientific), DIC (*N,N’*-Diisopropylcarbodiimide, Sigma Aldrich) and 1-hydroxybenzotriazole monohydrate (Sigma Aldrich). The following reagents: Et_3_N (Acros Organics), acetic anhydride (Fisher), pyridine (Sigma Aldrich) and DMF (Sigma Aldrich) were prepared according to literature procedures by distillation with calcium hydride and used immediately. Nuclear magnetic resonance spectra were measured on a *Bruker Avance III 500 MHz NMR Spectrometer* with TMS as an internal standard. MS spectra for water-insoluble compounds were collected using an Autoflex II MALDI-TOF mass spectrometer, and for water-soluble [60]fullerene derivatives by an MS electrospray ionization time-of-flight (ESI-microTOF) mass spectrometer, both instruments from Bruker Daltonics Inc (Fremont, CA). High resolution spectra were performed using Shimadzu IT-ToF LC-MS System and flash chromatography was performed using Isolera Flash Purification System The purity of all compounds was assessed using a Agilent1260 equipped with a DAAD detector at 260 nm, RP-column: Eclipse plus C18 (3,5 μm); flow 0.5 mL/min. The AFM experiments were performed using ambient pressure atomic force microscopy (AFM) NanoWizard3 from JPK Instruments. The surface topography and morphology were obtained using ultra-sharp tip (1 nm tip apex) well suitable for high resolution soft tapping mode. The data was analyzed using Gwyddion package^39^. The distributions of aggregates sizes were obtained by using masks on the topography maps, calculating the equivalent radius of observed.

Synthetic protocol for preparation of two [60]fullerene nanomaterials **GF1** and **GF2** is depicted on Fig. S1. The malonates (**1)** and **(2)** as well as glycofullerene **GF2** were synthesized according to previously described methodology^14^.

#### Synthesis of diserinol malonate acetate (**3**)

Diserinol malonate and its peracetylated version were synthesized on large scale by the modification of previously published synthetic protocol by Wharton and Wilson^40^. Briefly, 2-amino-1,3-propanediol (100 g, 1076 mmol) and dimethyl malonate (57.4 mL, 500 mmol) were dissolved in dry isopropanol, heated with vigorous stirring at 50 °C for 30 minutes and then stirred for 14 days at room temperature, under a nitrogen atmosphere. After that time, the white solid precipitated, was filtered off and washed with cold isopropanol. After recrystallization from isopropyl alcohol, the white solid was obtained and dried under lyophilization (m.p. 132 °C, yield 92%). Serinol malonate (25.0 g, 99.9 mmol) was suspended in pyridine (100 mL, 1240 mmol) at 0 °C, to which acetic anhydride (150 mL, 1590 mmol) was added dropwise over the course of an hour. After reaching 25 °C, dissolution occurred and the solution was stirred continuously for 48 hours. At 0 °C, methanol (50 mL, 1225 mmol) was added to quench remaining acetic anhydride and the solution was stirred for 1 hour, whereupon the solvents were removed *in vacuo* and the residue (yellow oil) was dissolved in dichloromethane and recrystallized over 12 hours in hexane. The precipitate as white crystals was collected by filtration, washed with hexanes and dried *in vacuo* (m.p. 91 °C, lit. 91–92 °C^40^).

#### Synthesis of [60]fullerene hexakis adduct (**GF1**)

C_60_ (720 mg, 1.00 mmol) was dissolved in freshly distilled toluene (800 mL) with CBr_4_ (497 mg, 1.5 mmol), and malonate **2** (461 mg, 1 mmol). At 25 °C, 1,8-diazabicyclo[5.4.0] undec-7-ene (DBU, 190 mg, 1.25 mmol) in toluene was added and the reaction was allowed to proceed for 3 hours at room temperature under a nitrogen atmosphere. Flash chromatography was used (dichloromethane: methanol, 95:5; silica: Mallinckrodt, 75–250 µm particles, 150 Å pore size) to isolate the product. Purified peracylated [60]fullerene monoadduct (65 mg, 0.055 mmol) was dissolved in freshly distilled toluene (150 mL) with CBr_4_ (365 mg, 1.1 mmol), and peracylated diserinol malonate (230 mg, 0.55 mmol). At 25 °C, 1,8-diazabicyclo[5.4.0] undec-7-ene (DBU, 100 mg, 0.66 mmol) in toluene was added slowly during 6 hours, and the reaction was allowed to proceed for 36 hours at room temperature under a nitrogen atmosphere. Flash chromatography was used (gradient from dichloromethane: methanol, 95:5 to 50:50; silica gel: Mallinckrodt, 75–250 µm particles, 150 Å pore size) to isolate the protected product. Yield: 57 mg (32%). Deprotection of acyl groups was performed in 18 mL of 1,4-dioxane with addiction of 2 mL of concentrated hydrochloric acid (36.5%) and then stirred at room temperature for 5 days. After that time, the final product was purified by dialysis of an aqueous solution of **7** using a cellulose membrane (molecular weight exclusion limit 1.0 kDa; Spectrum Labs, USA) up to the point where electrical conductivity of a purified glycofullerene became nearly equal to the conductivity of distilled water, and then lyophilized.

### Atomic force microscopy (AFM) studies

The investigated samples of glycofullerenes were transferred onto the atomically flat Si (001) surface in a form of small (10 µl) droplets. The droplets contained typically 0.1 mg/mL of sample mixed with water or the DMEM medium containing 12% FBS. The droplets were left under ambient pressure to evaporate excess of water and subsequently measured.

### Cell cultures

The cell cultures were prepared according to manufactures’ protocols as well as our previously developed methodology for anti-cancer agents and [60]fullerene nanomaterials^14,21^. Briefly, human pancreatic cell lines PANC-1 and AsPC-1 were purchased from Sigma Aldrich. The cells were grown as monolayer cultures in Dulbecco’s modified Eagle’s medium or RPMI-1640 medium in 75 cm^2^ flasks (Nunc). DMEM for PANC-1 cells was supplemented with 12% heat-inactivated fetal bovine serum (Sigma). RPMI-1640 for AsPC-1 cells was supplemented with 10% heat-inactivated fetal bovine serum. Both media also contained a mix of antibiotics −1% *v/v* of penicillin and streptomycin (Gibco). All of the cell lines were cultured under standard conditions at 37 °C in a humidified atmosphere with 5% CO_2_. All of the cell lines were subjected to routine mycoplasma testing using the PCR technique with specific *Mycoplasma* primers in order to ensure that there was no contamination.

### Cytotoxicity assay

Cytotoxicity studies for synthesized glycofullerenes were performed and evaluated by the activity of mitochondrial reductases (MTS assay), according to a well-established protocols previously described by our group for small-molecules^41,42^.

### Non-receptor tyrosine kinases inhibition *in vitro*

The inhibition of the tyrosine kinases by glycofullerenes were measured using Kinase Selectivity TK-2 profiling system and ADP-Glo assay (both reagents from Promega). This methodology was previously developed and described by our group for styrylquinazoline derivatives, and now extended and modified for [60]fullerene nanomaterials^21^. Before start experiments, the glycofullerenes being tested were dissolved in water or DMEM with 12% FBS to a concentration of 40 mg/mL (stock solution), which was used to prepare subsequent solutions in the concentration range of 0.05–2 mg/mL in a 1x Kinase Buffer. The results from these assays were presented as the percentage of the inhibition of tested kinases after treatment with the various concentrations of G**F1** and **GF2** nanomaterials. In addition, the inhibitory concentration (IC_50_) values were calculated using GraphPad Prism 7.0 (GraphPad Software, USA, https://www.graphpad.com).

### The mRNA expression of FYN and LCK kinases

A general method for analysis of mRNA expression of non-receptor kinases was described by our group for quinazoline derivatives and now extended and modified for [60]fullerene nanomaterials^21^. In short, after 24 h or 72 h treatment with tested glycofullerenes, total RNA was isolated from the pancreatic cells using TRIzol Reagent (Ambion) according to the supplier’s protocols. Single-stranded cDNA was synthesized from 4 μg of total RNA using a SuperScript IV Reverse Transcriptase kit with appropriate oligoprimers (dT)_20_ (both from Invitrogen). The quantitative Real-Time PCR was carried out in standard reaction volume of 20 μL, consisting of SsoAdvanced Universal SYBR Green Supermix (Biorad), primers mix (0.5 μM) and 1 μL of cDNA in CTX96 Touch Real-Time PCR Detection System (Biorad). The qRT-PCR reactions were carried out under the standard conditions: pre-denaturation (95 °C/30 sec), denaturation (95 °C/15 sec), annealing (temperature dependent on primers properties/30 sec), extension (72 °C/60 sec). The three last steps were repeated for 40 cycles. Data was interpretation and analyzed using the 2^−ΔΔCT^ method as described by Livak and Schmittgen^43^. The sequences of *FYN*, *LCK* and *ACTB* (reference gene) primers were designed in Primer 3 and were bought from Sigma Aldrich (see Table S1**)**.

### Basal level of the mRNA expression of GLUT-1 in pancreatic cells

Total RNA was isolated from PANC-1 and AsPC-1 cells using TRIzol Reagent (Ambion) according to the manufacturer’s instructions. The cDNA synthesis and qRT-PCR reaction were performed as were described above. Target and reference primer pair sequences were designed in Primer 3 and were purchased from Sigma Aldrich (Table S2). Data was analyzed based on a comparison of the expression of the target genes to a reference gene – *GAPDH*, using the 2^−ΔΔCT^ method. The experiments were performed at least four times.

### Western blotting

Immunoblotting experiments were carried out using a well-established protocols previously described by our group for small-molecules^35^. Cellular lysates were collected after 24 h or 72 h treatment with glycofullerenes (1 mg/mL) in PANC-1 and AsPC-1 cell lines. The quantitation of protein was measured using a Micro BCA Protein Assay Kit (Thermo Scientific) according to the supplier’s manuals. The proteins (20 μg) were separated on SDS-Page gels and electrotransferred onto 0.2 µm pore-size nitrocellulose membranes. After blocking step, the nitrocellulose membranes with proteins were incubated with specific primary antibodies: Fyn, Lck, p21, p53, HO-1, vinculin and GAPDH (all diluted 1:1000) overnight, then washed and incubated with horseradish peroxidase-conjugated secondary antibodies (1:1000 dilution). All primary and secondary antibodies were bought from CellSignaling. Finally, the horseradish peroxidase activity signal were detected using SuperSignal West Pico Chemiluminescent Substrate (Thermo Scientific) in ChemiDoc XRS + System (BioRad). The densitiometric analysis was performed as described elsewhere^35^.

### Cell cycle analysis

Cell cycle analysis was carried out according to a well-established protocols previously described by our group for small-molecules^35^. Briefly, PANC-1 and AsPC-1 cells were seeded according to standard procedures. Then, freshly prepared solutions of glycofullerenes at 1 mg/mL concentration were added in 3 cm Petri dishes (Nunc). The changes of cell cycle of pancreatic cells were determined after a 24 hour treatment with tested glycofullerenes using a Muse Cell-Cycle Kit (Millipore) according to the manufacturer’s protocols.

### Autophagy induction assay

PANC-1 and AsPC-1 cells were seeded in 96-well plates (Nunc) at a density of 20,000 cells/well (for 24 h assay) and 10,000 cells/well (for 48 h assay) and incubated at 37 °C for 24 hours. Then, the medium was removed and freshly prepared solutions of glycofullerenes at 1 mg/mL concentration were added and the cells were incubated for 24 or 48 hours. As positive control, cells were treated with Imatinib (25 µM) for 24 hours. After treatment, assays were performed using a MuseAutophagy LC3-antibody based kit (Millipore) according to the manufacturer’s instructions. Briefly, cells were collected, washed with cold HBSS and centrifuged at 300 g for 5 min. Afterwards, cells were resuspended in 95 μL of 1X Autophagy Reagent B with 5 μL of Anti-LC3 Alexa Fluor555 antibody and incubated for 30 minutes on ice in the dark. After this time, the cells were centrifuged and resuspended in 200 μL of 1X Assay Buffer. Then, the cells immediately were processed for autophagy induction analysis using a Muse Cell Analyzer (Millipore). Autophagy induction ratio were calculated based on the ratio between the target sample fluorescence versus the control sample. The experiments were performed at least three times.

### ROS formation

The ROS measurements were performed according to protocols described previously by our group^35^. In short, PANC-1 and AsPC-1 cells were seeded according to standard procedures. Prior the experiments, the fresh solutions of glycofullerenes at 1 mg/mL concentration were prepared, which were then transferred into plate and incubated for 9, 12 and 24 hours in a kinetic experiment. For quenching reactive oxygen species, Neocuproine at 50 μM concentration was added. The ROS formation was determined using a CellROX Green Reagent (Molecular Probes) according to the supplier’s protocols.

### Protein corona assay

Preparation of protein corona and assessments of nanomaterials interactions with FBS proteins were performed according to Strojan *et al*.^44^, with some modifications. In short, **GF2** glycofullerene was prepared to a final concentration 2 mg/mL or 1 mg/mL in the 100% FBS solution and 1 mg/mL in RPMI medium supplemented with 10% FBS. Afterwards, FBS-**GF2** mixtures were incubated for 2–3 h in 37 °C. Then, the samples (FBS-**GF2**) and control (100% FBS) were centrifuged at 15,000 x *g* for 20 minutes at 4 °C, supernatants were removed and pellets were re-suspended in 1 mL cold PBS without CaCl_2_ and MgCl_2_. This washing procedure was repeated three times to remove non-bounded proteins. After last centrifugation, samples were re-suspended in NuPAGE LDS Sample Buffer, then vortexed and heated for 5 min at 95 °C. At this stage, FBS-**GF2** complexes were broken down, and **GF2** were removed by centrifugation at 15,000 × g for 20 min. The equal amounts of the remaining proteins, which interacted with glycofullerene were separated on SDS-PAGE gels. After electrophoresis, the gels were stained with Coomassie Stain G-250 reagent for 30 min. The image were acquired using a ChemiDoc XRS + System (BioRad) and densitometric analysis was performed as described elsewhere^35^.

### Statistical analysis

Results are expressed as the mean ± standard deviation (SD) from at least three independent experiments. Statistical differences in the expression of genes and proteins, autophagy induction and progression of cell cycle were calculated using the one-way ANOVA with a Bonferroni post-hoc test. A p-value of 0.05 or less was considered to be statistically significant. GraphPad Prism 7.0 software (GraphPad Software, USA, https://www.graphpad.com) was used for analysis.

## Supplementary information

Supplementary Information.
