# Supplementary material for: Glycofullerenes as non-receptor tyrosine kinase inhibitors- towards better nanotherapeutics for pancreatic cancer treatment
Source: Sci Rep. 2020 Jan 14;10:260. doi: 10.1038/s41598-019-57155-7 (PMC6959220; doi:10.1038/s41598-019-57155-7)

Supplementary Materials for

Glycofullerenes as non-receptor tyrosine kinase inhibitors- towards better nanotherapeutics for pancreatic cancer treatment

Maciej Serda[*1], Katarzyna Malarz[2], Anna Mrozek-Wilczkiewicz[2], Marcin Wojtyniak[2], Robert Musioł[1], Steven A. Curley[3]

[1]*Institute of Chemistry, University of Silesia in Katowice, Katowice, 40-006, Poland*

[2]*Institute of Physics and Silesian Center for Education and Interdisciplinary Research, University of Silesia in Katowice, 75 Pułku Piechoty 1A, 41-500 Chorzów, Poland*

[3]*CHRISTUS Trinity Mother Frances Oncology Institute, Tyler, TX 7570, USA*

**Author for correspondence: Dr. Maciej Serda (maciej.serda@us.edu.pl)*

S1 CHEMISTRY

- Synthetic protocol performed for glycofullerenes **GF1** and **GF2**
- Dynamic Light scattering (DLS) and zeta potential measurements
- AFM measurements for **GF1** and **GF2**
- MALDI TOF mass spectrometry of peracetylated **GF1**
- ESI TOF mass spectrometry of **GF1**

S2 BIOLOGY

- Analysis of the mRNA expression of *FYN* and *LCK* kinases
- Basal level of the mRNA expression of *GLUT-1* in pancreatic cells
- mRNA expression of GLUT-1 in PANC-1 and AsPC-1 cells
- Generation of ROS by **GF1** and **GF2** in PANC-1 and AsPC-1 cells
- Analysis of **GF2** interactions with FBS using SDS-PAGE electrophoresis

Figure S1

Synthetic protocol performed for glycofullerenes **GF1** and **GF2**

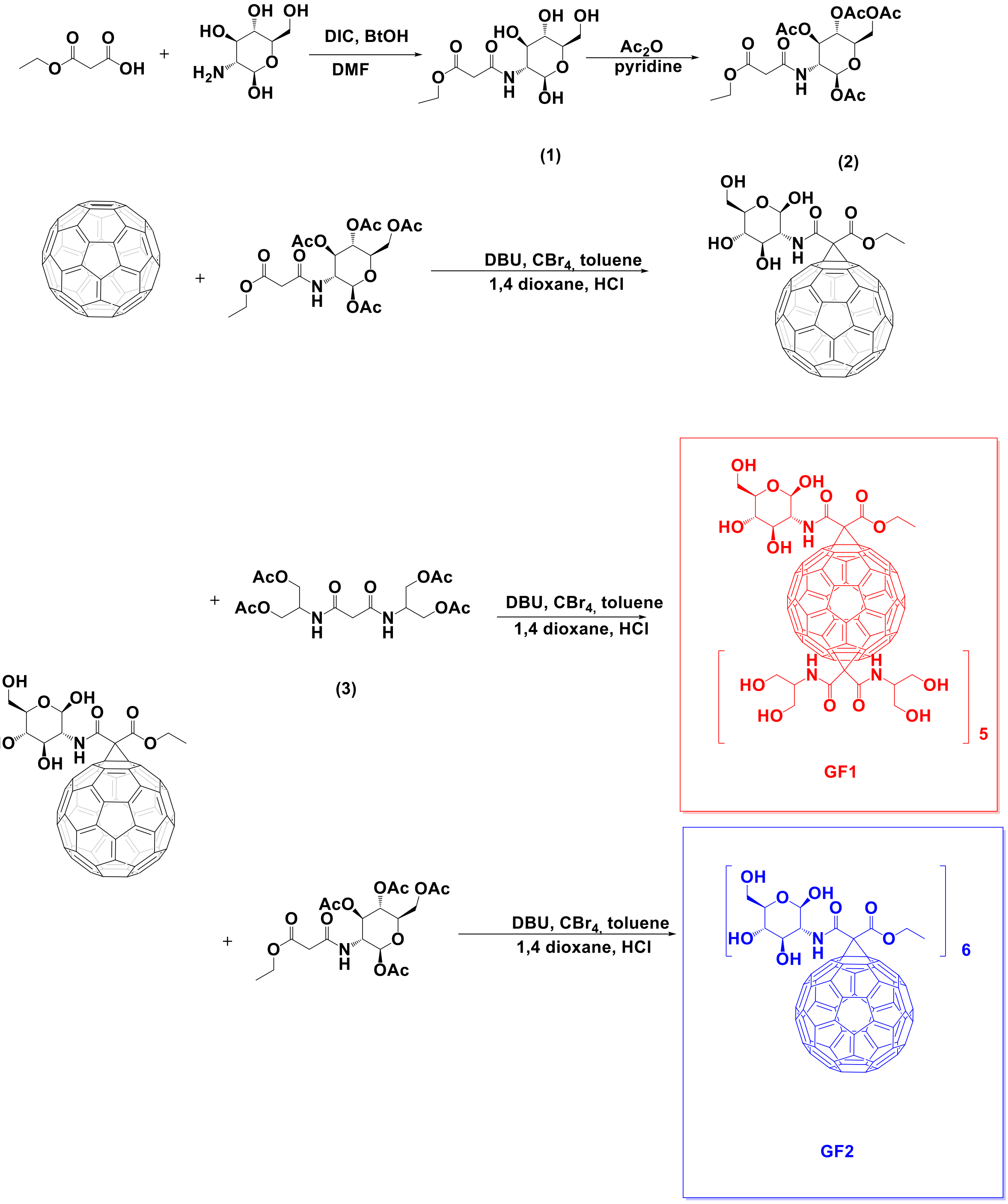

Dynamic light scattering, Zeta-potential Analysis

Dynamic light scattering analysis (DLS) was performed using a Malvern Nano-ZS90 zetasizer (Malvern Instruments Ltd., Worcestershire, United Kingdom) with detection limit ca 1 nm. The Nano-ZS employs noninvasive backscatter (NIBS) optical technology and measures real-time changes in intensity of scattered light. The samples were illuminated by a 633 nm He-Ne laser and the scattered light was measured at an angle of 173° using a photo-diode. The size distribution of the glycofullerenes was calculated from the particle diffusion coefficient according to the Stokes-Einstein equation.

Figure S2

DLS analysis of DMEM containing 12 % of FBS.

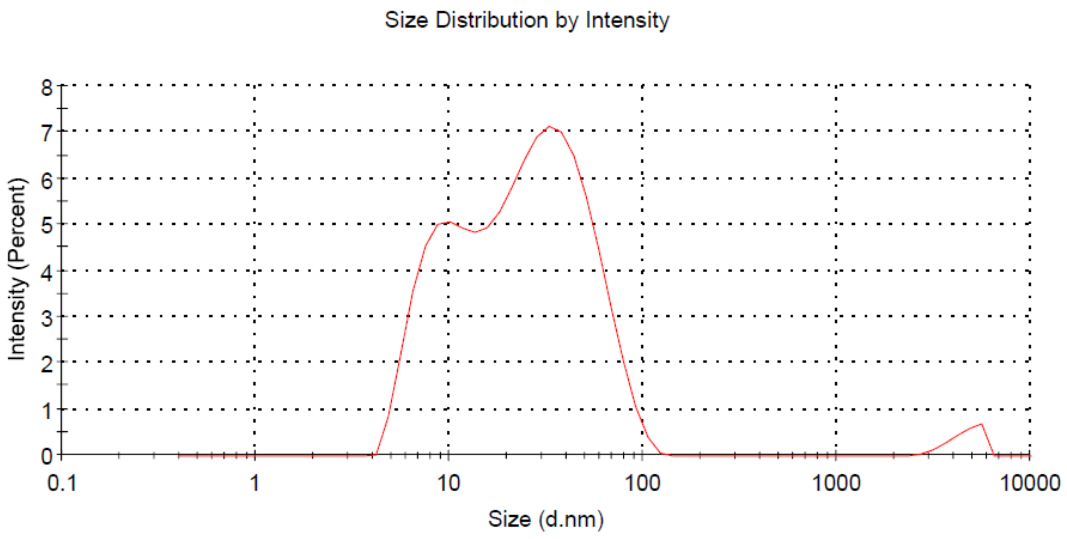

	Size (d.n...	% Intensity:	St Dev (d.n...
**Peak 1:**	35.90	68.4	18.91
**Peak 2:**	9.410	29.6	2.598
**Peak 3:**	4629	2.1	817.6

Figure S3

DLS of **GF1** in distilled water (c=0.001 mg/mL)

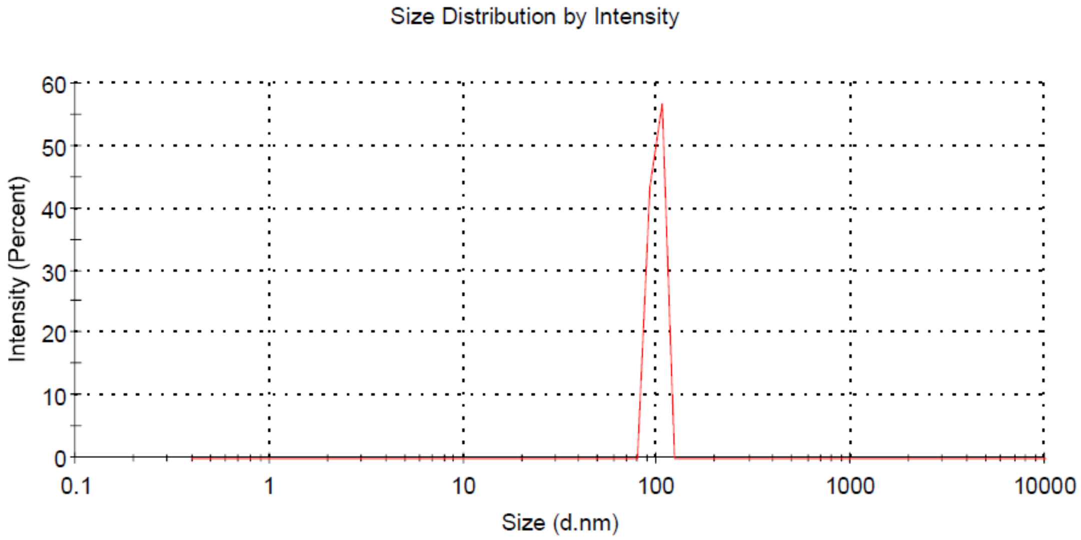

	Size (d.n...	% Intensity:	St Dev (d.n...
**Peak 1:**	99.46	100.0	7.150

Figure S4

Zeta potential measurement of **GF1** in distilled water (c=0.001 mg/mL).

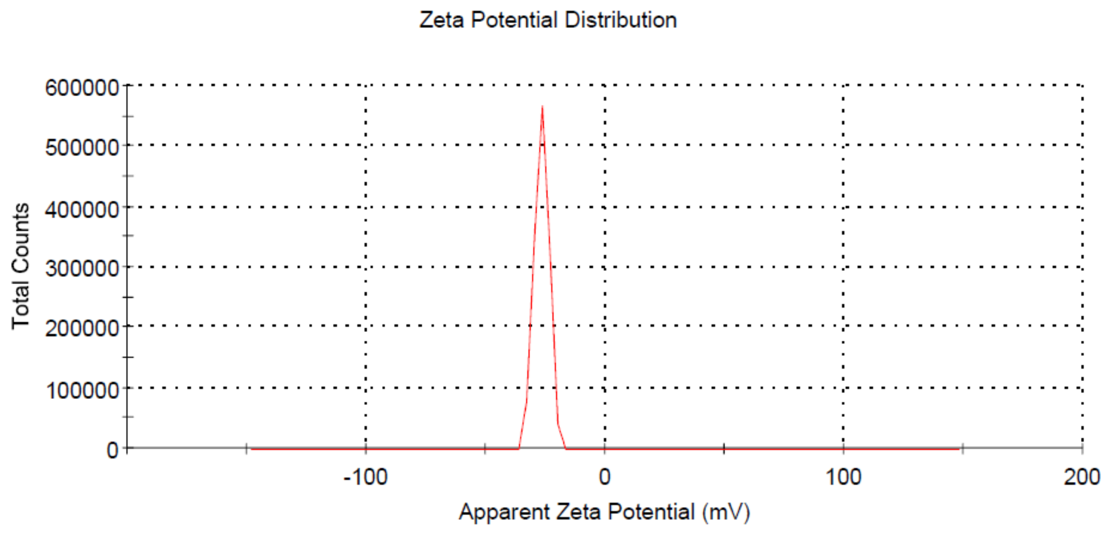

	Mean (mV)	Area (%)	St Dev (mV)
**Peak 1:**	-26.7	100.0	3.02

Figure S5

DLS of **GF1** in DMEM containing 12 % FBS (c=0.001 g/L)

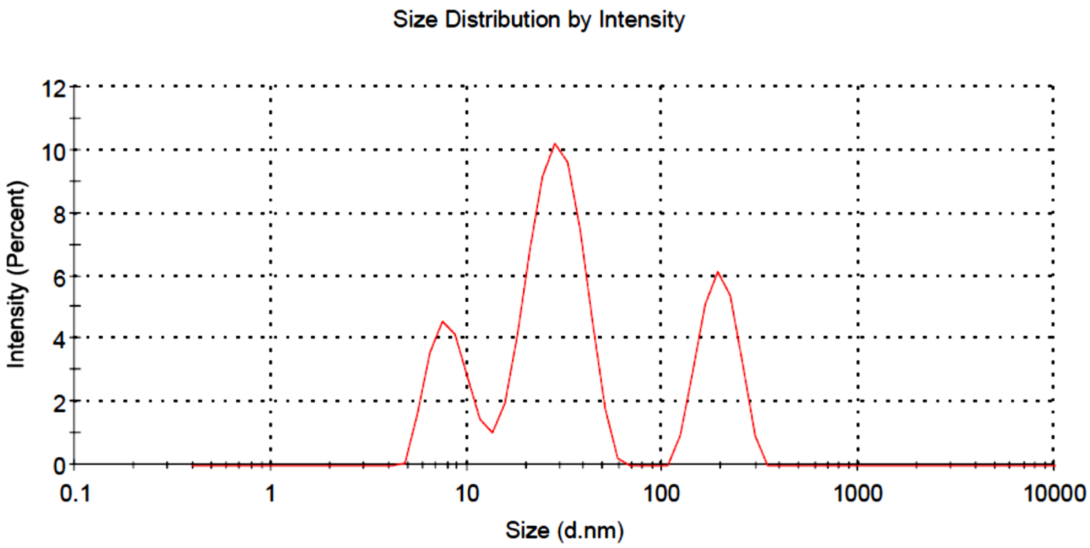

	Size (d.n...	% Intensity:	St Dev (d.n...
**Peak 1:**	29.39	56.4	8.864
**Peak 2:**	195.4	24.5	41.69
**Peak 3:**	8.435	19.2	2.060

Figure S6

The AFM studies of glycofullerenes **GF1** and **GF2**

A- surface topography for the single crystal Si (001) acting as a substrate for the investigated glycofullerenes; **B-** smaller aggregates of **GF1** in water with the average diameter equal to 19.24 nm (c= 0.1 mg/mL); **C-** larger aggregates of **GF1** in water with the average diameter equal to 285.1 nm (c= 0.1 mg/mL); **D-** smaller aggregates of **GF2** in water with the average diameter equal to 17.68 nm (c= 0.1 mg/mL); **E-** larger aggregates of **GF2** in water with the average diameter equal to 268.5 nm (c= 0.1 mg/mL); **F-** sample of **GF1** diluted in DMEM cellular medium containing 12% FBS.

A

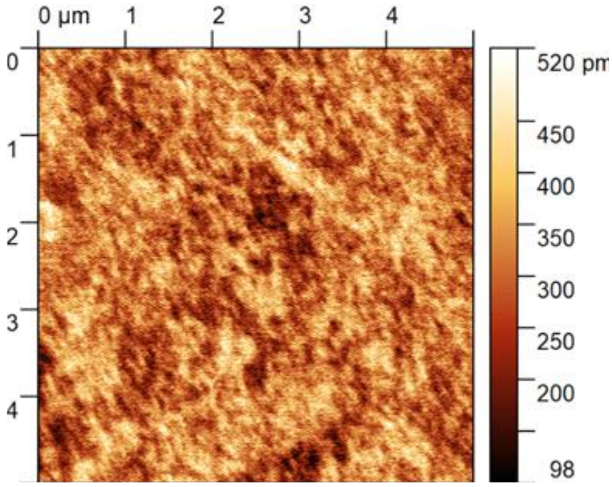

B

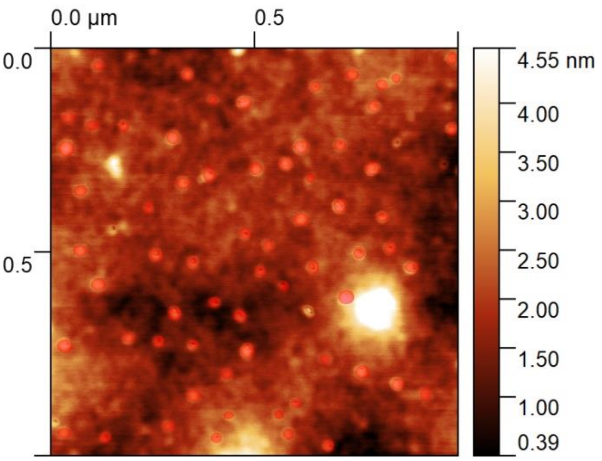

C

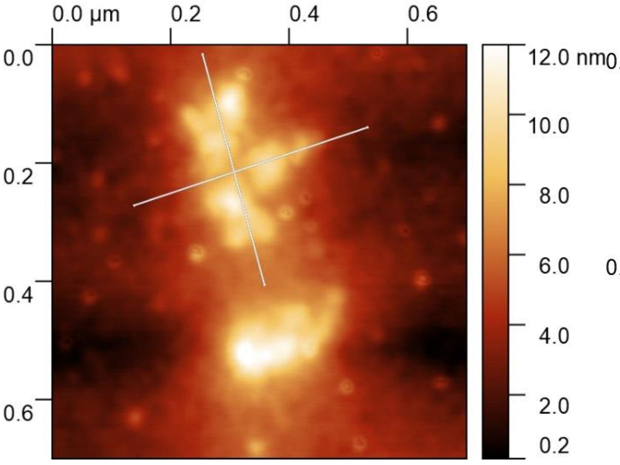

D

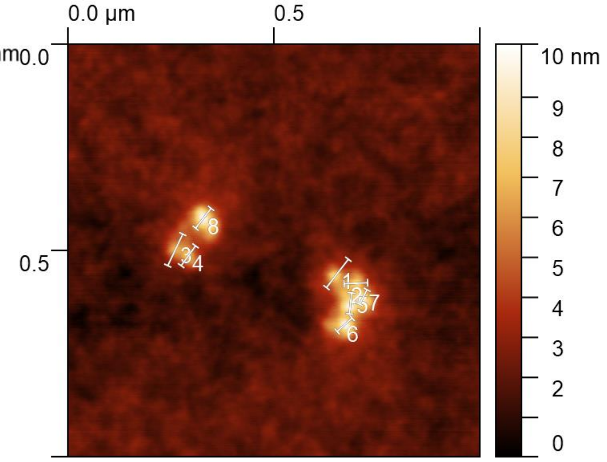

E

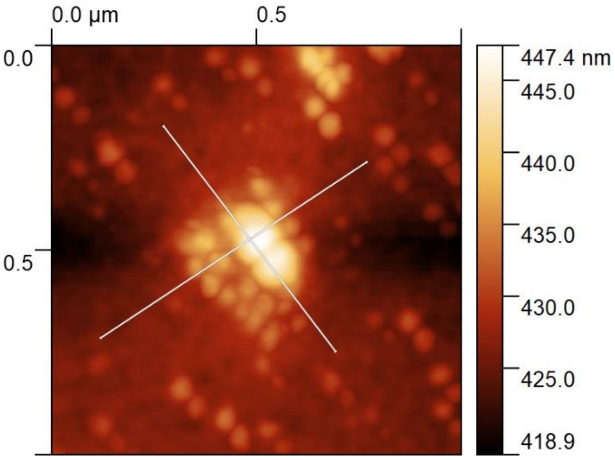

0.0 µm
0.5
0.0
0.5
447.4 nm
445.0
440.0
435.0
430.0
425.0
418.9

F

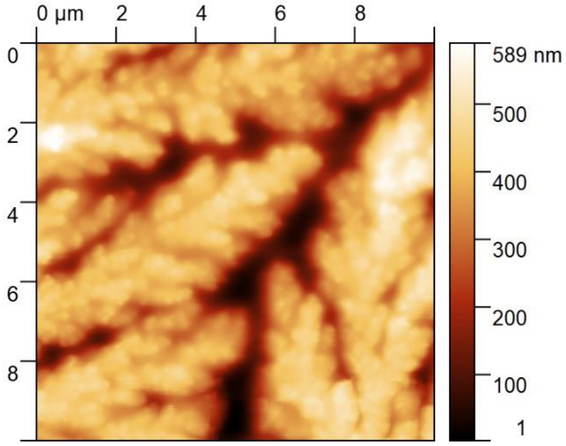

0 µm
2
4
6
8
0
2
4
6
8
589 nm
500
400
300
200
100
1

Figure S7

MALDI TOF (POSITIVE) spectrum of acetyl protected **GF1**

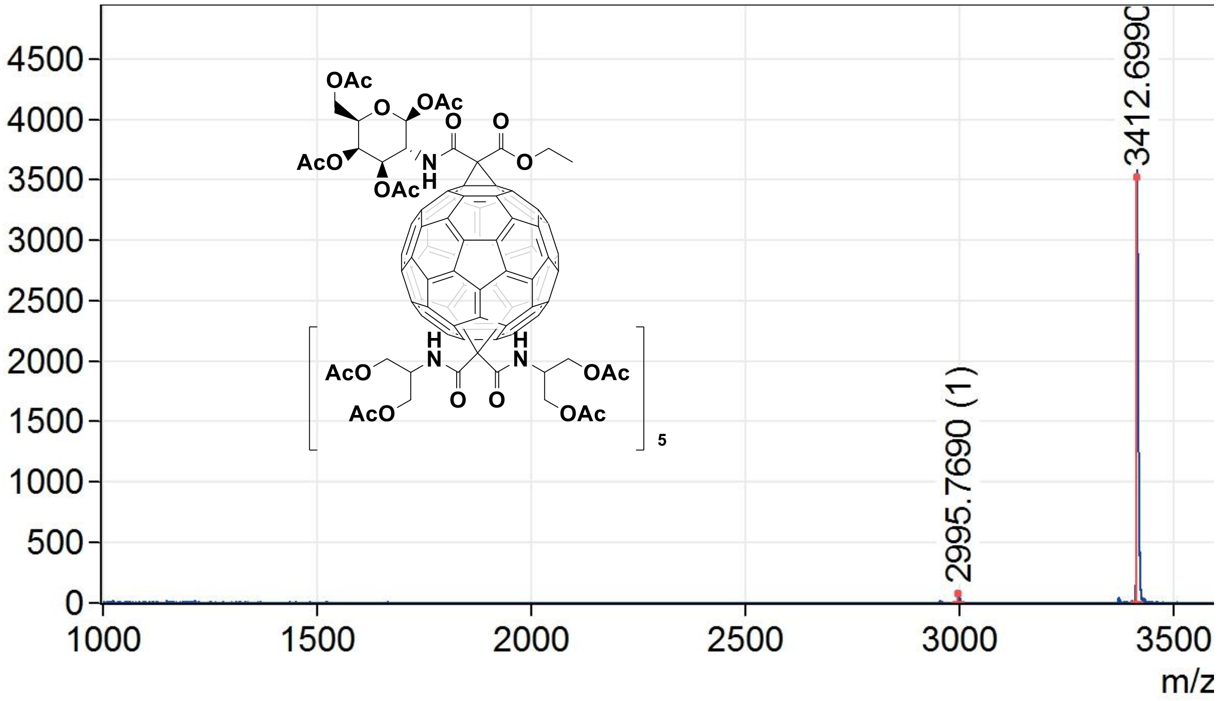

Figure S8

ESI-MICRO-TOF (POSITIVE IONIZATION 200 mV) OF **GF1**

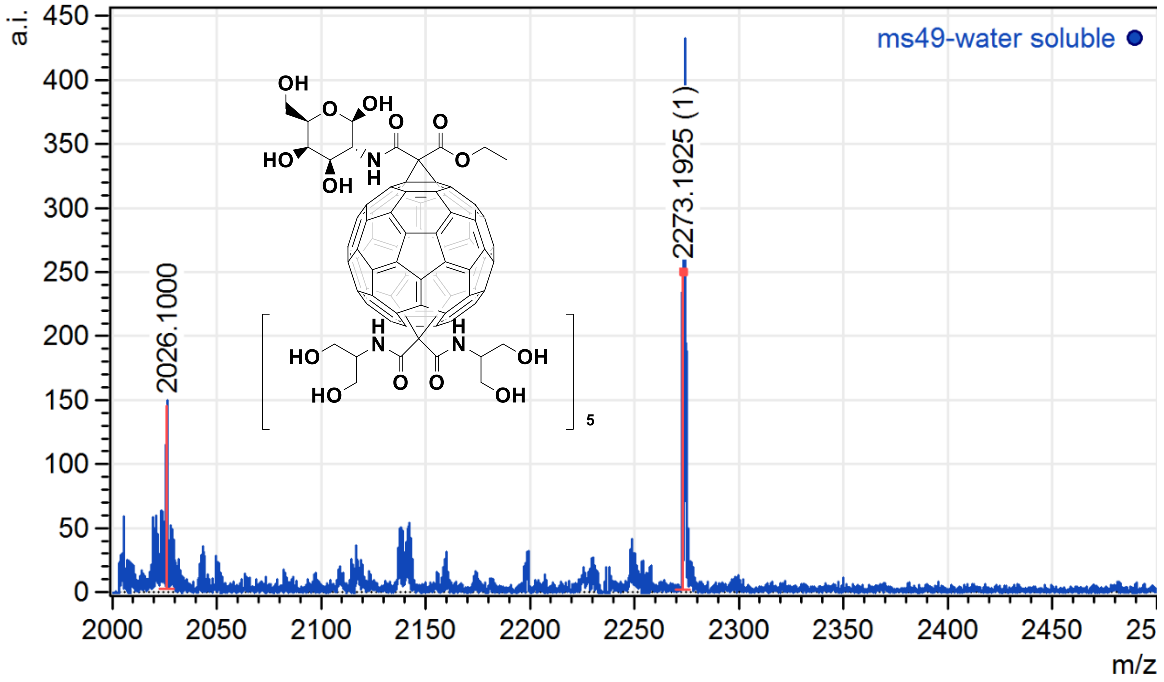

BIOLOGY

Table S1. Primer pair sequences that were used to determine the mRNA expression of *FYN*, *LCK* and *ACTB*

Gene	GenBank accession no.	Forward primer	Reverse primer
FYN	NM_002037	5'-GTAGTCATGGCAACCCGCTA-3'	5'-ACAACCCCCACCCTCATTTC-3'
LCK	NM_001042771	5'-AGCTTTTCTGTGGCTGGTGA-3'	5'-CATTTCGGATGAGCAGCGTG-3'
ACTB	NM_001101	5'-CTCGCCTTTGCCGATCC-3'	5'-GCTGGGGTGTTGAAGGTCTC-3'

Table S2. Primers sequences that were used to determine the mRNA expression of *GLUT-1* and *GAPDH*

Gene	GenBank accession no.	Forward primer	Reverse primer
GLUT-1	NM_006516	5'-AAGGTGATCGAGGAGTTCTACA-3'	5'-ATGCCCCCAACAGAAAAGATG-3'
GAPDH	NM_002046	5'-GAGTCAACGGATTTGGTCGTA-3'	5'-GCCCCACTTGATTTTGGAG-3'

Figure S9

mRNA expression of *GLUT-1* in PANC-1 and AsPC-1 cells

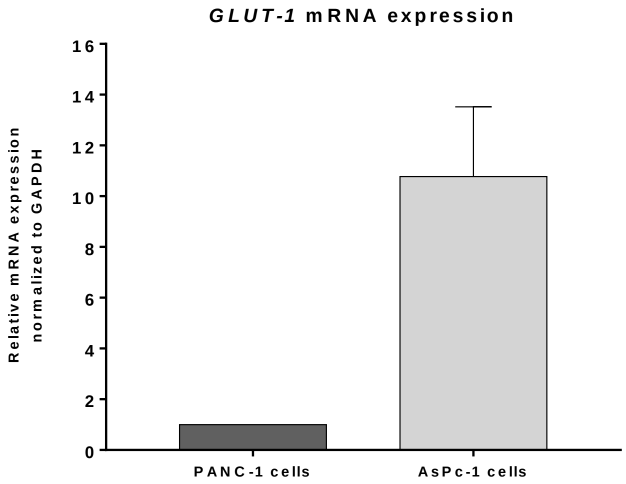

Figure S10

Impact of glycofullerenes and Neocuproine (quencher) on the generation of reactive oxygen species (ROS) in PANC-1 (A) and AsPC-1 (B) cells.

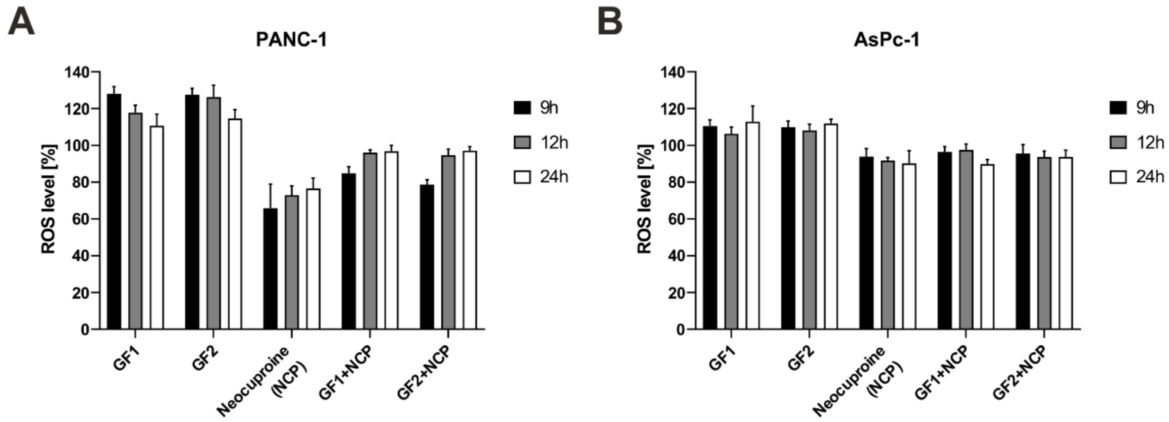

Figure S11

A-Analysis of **GF2** interactions with FBS proteins using SDS-PAGE electrophoresis; **B-**Densitometric analysis of 60 kDa, 110 kDa and 160 kDa protein bands. Data was normalized to 100% FBS, which was used as control.

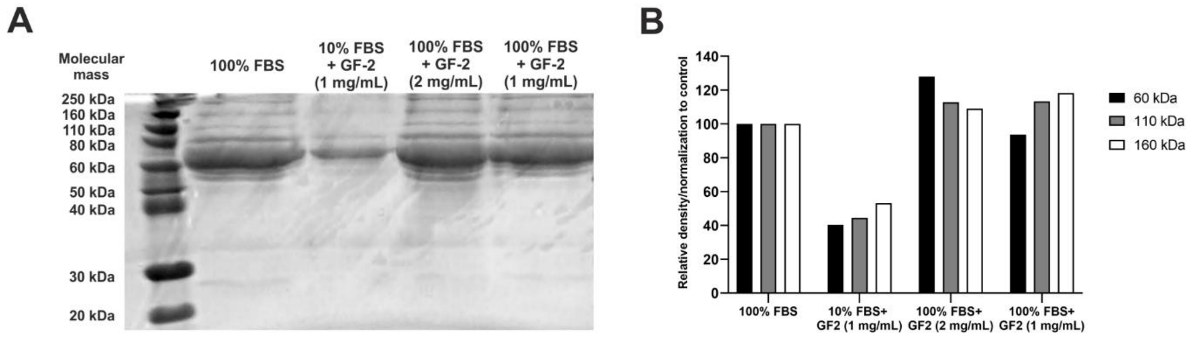

Supplement: Supplementary file 1 — Supplementary Information. [file 41598_2019_57155_MOESM1_ESM.pdf]
